# Supplementary material for: Association between oral microbiome and seven types of cancers in East Asian population: a two-sample Mendelian randomization analysis
Source: Front Mol Biosci. 2023 Nov 21;10:1327893. doi: 10.3389/fmolb.2023.1327893 (PMC10702768; doi:10.3389/fmolb.2023.1327893)
Supplement: Supplementary file 1 [file DataSheet1.ZIP › Supplementary Materials/MR plots for tongue/tongue═╝/Ovarian cancer/pheno.173_to_ovarian cancer_leave_one_out.pdf]

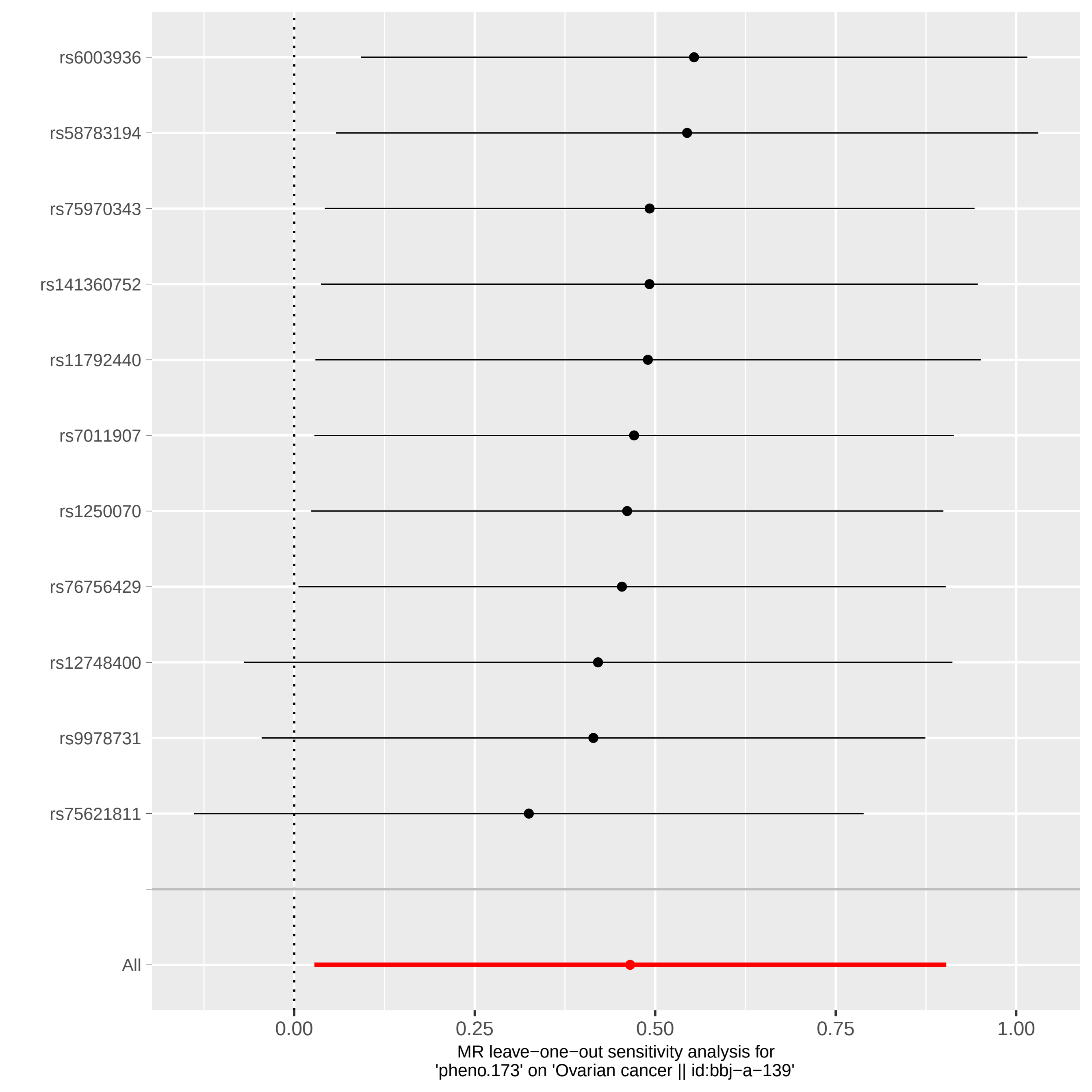

rs6003936
rs58783194
rs75970343
rs141360752
rs11792440
rs7011907
rs1250070
rs76756429
rs12748400
rs9978731
rs75621811
All
0.00
0.25
0.50
0.75
1.00
MR leave−one−out sensitivity analysis for
'pheno.173' on 'Ovarian cancer || id:bbj−a−139'
